# Data on occurrence of perfluoroalkyl substances in influents and effluents collected from different wastewater treatment plants in Latvia

**DOI:** 10.1016/j.dib.2022.108228

**Published:** 2022-04-29

**Authors:** Dzintars Zacs, Elina Pasecnaja, Vadims Bartkevics

**Affiliations:** a Institute of Food Safety, Animal Health and Environment „BIOR”, Lejupes iela 3, Riga LV-1076, Latvia; b University of Latvia, Faculty of Chemistry, Jelgavas street 1, Riga LV-1004, Latvia

**Keywords:** Perfluorocarboxylic acid, Perfluorosulfonic acid, Wastewater, Occurrence

## Abstract

The data set provided in this paper contains occurrence data of 17 perfluoroalkyl substances (PFAS) (including 10 perfluorinated carboxylic acids and 7 perfluorinated sulfonic acids) in influent and effluent samples collected from 43 wastewater treatment plants (WWTPs) located in different cities in Latvia. Samples were collected in the period June-July 2021. In each WWTP one influent and one effluent sample were collected on the same day. Extraction and clean-up of the samples were performed using solid phase extraction (SPE) on a weak-anion SPE phase. Observed extracts were analysed using high performance liquid chromatography coupled with Orbitrap high resolution mass spectrometry (HPLC-Orbitrap-MS) on the content of selected PFAS representatives. The collected data are with fundamental scientific value and can be applied for local data analysis. The data set is useful for the estimation of overall background levels of PFAS and evaluation of local city WWTPs wastewater remediation efficiency towards the removal of PFAS contamination.

## Specifications Table

**Table:** 

Subject	*Environmental Science*
Specific subject area	*Emerging Contaminants in the environment*
Type of data	*Tables* *Figure*
How the data were acquired	*High performance liquid chromatography coupled with Orbitrap high resolution mass spectrometry (Thermo Scientific UltiMate 3000 HPLC coupled with Thermo Scientific Orbitrap Q-Exactive mass spectrometer). Xcalibur™ Software.*
Data format	Raw; Analysed
Description of data collection	*Wastewater samples were collected into 1* *L brown coloured glass bottles and transferred to the laboratory at + 4* °*C. Samples were stored at +4* °*C and extracted within seven days of receipt. Samples were extracted by weak-anion exchange solid phase extraction and subjected to HPLC-Orbitrap-MS analysis.*
Data source location	*Institution: Institute of Food Safety, Animal Health and Environment “BIOR”*• City: Riga• Country: Latvia• Latitude and longitude for monitoring points are presented in Table 1
Data accessibility	The data are available within this article

## Value of the Data

• Perfluorinated substances transport and spread in the environment through WWTPs are of increasing concern, with a lack of data available in Latvia. These data offer a comprehensive overview of the occurrence of 17 perfluorinated carboxylic and sulfonic acids in influents and effluents collected from different WWTPs in Latvia.
• The data can be valuable for researchers studying contaminant transfer into the environment through the WWTPs.
• These data may be used by local authorities, policy makers and researchers to improve the environmental quality standards. These data may be valuable in the optimisation of WWTP operation conditions.
• These data may contribute to the existing worldwide need of monitoring data for PFAS to support future prioritisation of relevant compounds and the development of guidelines by national and international authorities.

## 1 Data Description

**Fig. 1:**
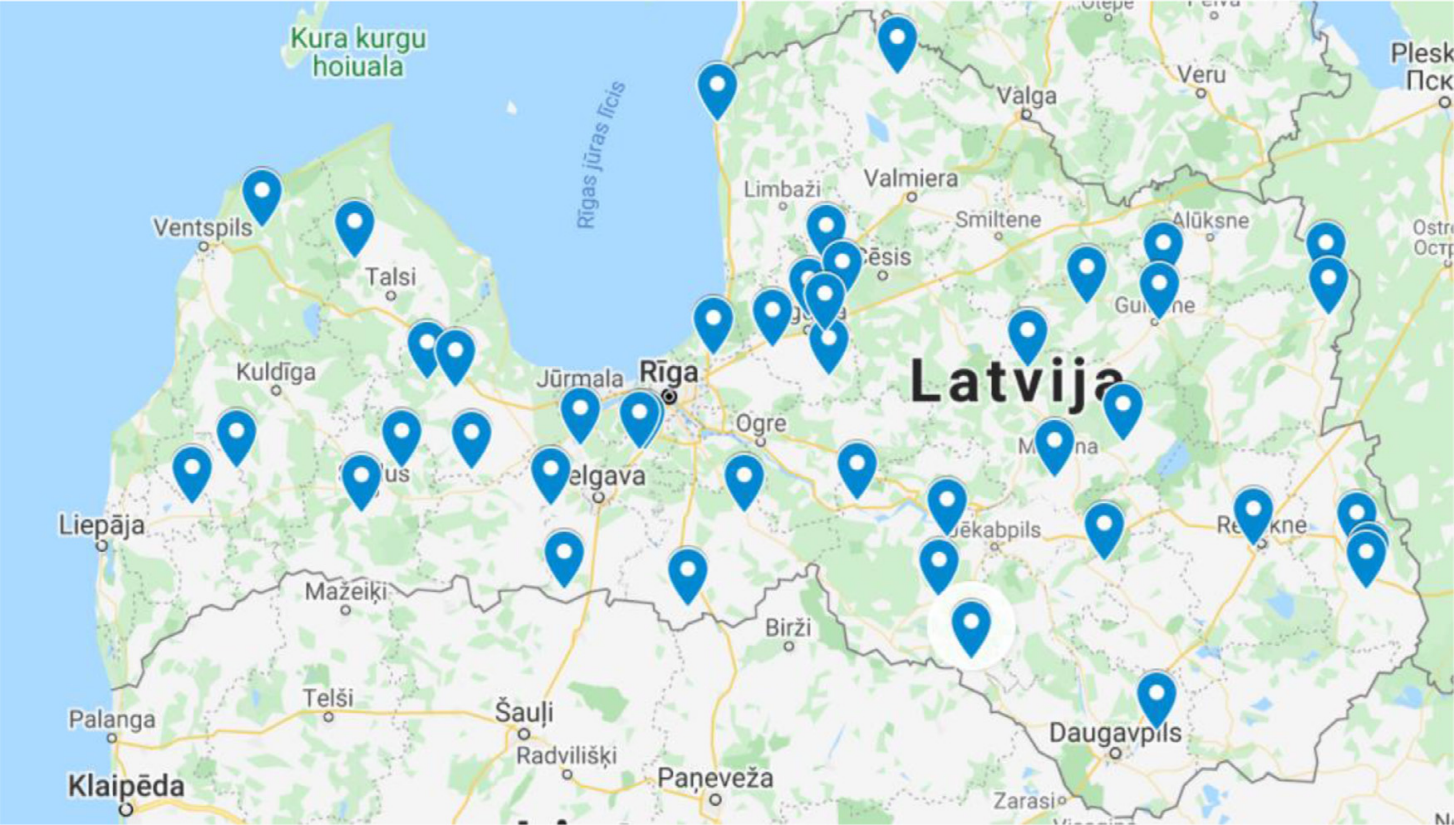
The distribution of sampling locations throughout Latvia.

**Fig. 2:**
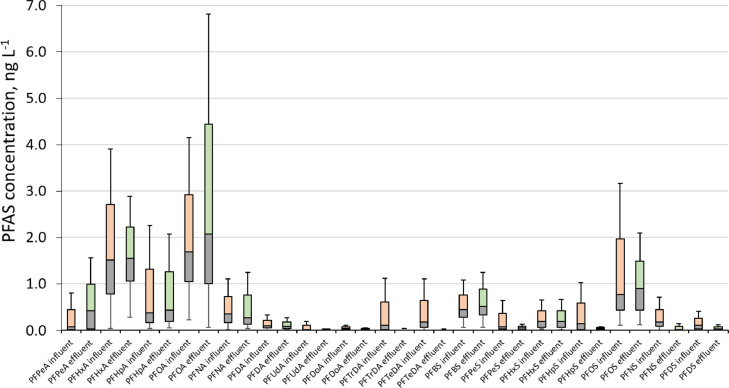
Boxplots of the concentrations of selected PFAS in analysed influents and effluents.

The presented data were obtained during a comprehensive monitoring study on the occurrence of 17 PFAS in influent and effluent samples collected from 43 WWTPs located in different regions of Latvia. Results are expressed as nanogram per liter concentrations (ng L^−1^). Data presented include (1) mapping of sampling locations in different regions of Latvia (Fig. 1); (2) datasets with concentrations of selected PFAS in influent wastewater collected before any treatment and concentrations in effluent wastewater collected immediately before the discharge from WWTP (Table 1); (3) constructed boxplots for datasets (Fig. 2).

**Table 1:** Dataset with concentrations of selected PFAS in influent wastewater collected before any treatment and concentrations in effluent wastewater collected immediately before the discharge from WWTP.

**Sampling location**	**Coordinates**	**PFPeA**	**PFHxA**	**PFHpA**	**PFOA**	**PFNA**	**PFDA**	**PFUdA**	**PFDoA**	**PFTrDA**	**PFTeDA**	**PFBS**	**PFPeS**	**PFHxS**	**PFHpS**	**PFOS**	**PFNS**	**PFDS**
WWTP "Piebalgas", Vecumnieki district, influent	56.60057, 24.49447	1.14	2.69	1.19	3.06	1.21	0.79	0.15	<LOQ	<LOQ	<LOQ	0.57	0.14	0.25	0.05	0.22	<LOQ	<LOQ
WWTP "Piebalgas", Vecumnieki district, effluent		0.99	2.86	1.11	4.70	0.84	0.45	0.01	<LOQ	<LOQ	<LOQ	0.33	<LOQ	0.10	0.05	0.46	<LOQ	0.82
WWTP "Uzvara", Bauska district, influent	56.31715, 24.19518	0.30	0.14	0.08	2.04	0.11	0.01	<LOQ	<LOQ	<LOQ	<LOQ	0.22	<LOQ	<LOQ	<LOQ	0.11	0.03	0.11
WWTP "Uzvara", Bauska district, effluent		0.45	0.29	0.12	0.75	0.11	0.03	<LOQ	<LOQ	<LOQ	<LOQ	0.29	<LOQ	<LOQ	<LOQ	0.35	<LOQ	<LOQ
WWTP "Nākotne", Jelgava region influent	56.61932, 23.44394	1.01	2.40	2.74	1.92	0.81	0.03	<LOQ	<LOQ	<LOQ	<LOQ	0.31	<LOQ	<LOQ	<LOQ	0.46	<LOQ	<LOQ
WWTP "Nākotne Jelgava region effluent		1.20	1.10	2.09	0.82	0.76	0.08	0.01	<LOQ	<LOQ	<LOQ	0.48	<LOQ	<LOQ	0.02	0.40	<LOQ	<LOQ
Vilce village WWTP, Jelgava region, influent	56.37061, 23.52569	1.09	4.06	0.57	5.02	0.29	0.10	<LOQ	<LOQ	<LOQ	<LOQ	0.53	<LOQ	0.04	0.20	0.29	<LOQ	0.68
Vilce village WWTP, Jelgava region, effluent		0.90	2.16	0.43	1.62	0.24	0.14	<LOQ	<LOQ	<LOQ	<LOQ	0.53	<LOQ	<LOQ	0.02	0.34	<LOQ	0.07
Kalnciems village WWTP, Kalnciems, influent	56.80218, 23.60559	0.79	2.62	8.50	1.32	0.54	0.19	0.02	<LOQ	<LOQ	<LOQ	1.02	<LOQ	0.05	<LOQ	0.40	<LOQ	0.36
Kalnciems village WWTP, Kalnciems, effluent		1.60	1.29	2.54	64.1	<LOQ	<LOQ	<LOQ	<LOQ	<LOQ	<LOQ	0.69	<LOQ	<LOQ	<LOQ	<LOQ	<LOQ	<LOQ
Ezernieki WWTP, Ezernieki, influent	56.81207, 26.56585	0.01	1.20	0.15	0.89	0.24	0.12	<LOQ	0.04	<LOQ	<LOQ	0.22	0.53	0.21	<LOQ	0.43	0.45	<LOQ
Ezernieki WWTP, Ezernieki, effluent		0.03	0.83	0.19	3.71	0.20	0.05	<LOQ	<LOQ	<LOQ	<LOQ	0.51	0.01	<LOQ	0.02	1.29	0.02	<LOQ
Naujene WWTP, Naujene parish, influent	55.94411, 26.7545	0.07	2.77	1.39	2.19	0.52	0.12	<LOQ	<LOQ	<LOQ	<LOQ	1.04	0.40	<LOQ	<LOQ	1.97	0.18	0.10
Naujenes WWTP, Naujenes parish, effluent		0.12	2.19	0.87	2.68	0.69	0.17	<LOQ	<LOQ	<LOQ	<LOQ	0.94	0.08	0.03	<LOQ	1.43	0.15	0.03
Rudzāti WWTP, Rudzāti, influent	56.45533, 26.47109	0.01	0.82	0.22	1.50	0.19	0.09	<LOQ	<LOQ	<LOQ	<LOQ	0.39	0.01	0.06	<LOQ	0.72	<LOQ	<LOQ
Rudzāti WWTP, Rudzāti, effluent		0.02	0.50	0.29	2.05	0.07	<LOQ	<LOQ	<LOQ	<LOQ	<LOQ	0.55	0.00	0.15	<LOQ	0.47	0.02	<LOQ
Viesīte WWTP, Viesīte, influent	56.34486, 25.56032	0.06	3.71	0.26	1.69	0.46	0.10	<LOQ	<LOQ	<LOQ	<LOQ	0.60	0.08	0.58	<LOQ	1.69	<LOQ	<LOQ
Viesīte WWTP, Viesīte, effluent		0.01	1.72	0.19	6.48	0.66	0.18	<LOQ	<LOQ	<LOQ	<LOQ	1.09	0.05	0.02	<LOQ	1.15	0.02	<LOQ
Aknīste WWTP, Aknīste, influent	56.15973, 25.74428	0.13	4.41	0.44	3.08	2.02	0.11	<LOQ	<LOQ	<LOQ	<LOQ	0.47	0.07	0.15	<LOQ	0.77	0.15	0.12
Aknīste WWTP, Aknīste, discharge		0.12	2.31	0.09	7.43	2.73	0.24	<LOQ	0.09	<LOQ	<LOQ	0.73	0.34	0.41	<LOQ	1.48	0.13	0.07
Straupe PKS, "Pienotava", Straupe parish, influent	57.34699, 24.9477	0.03	2.42	0.08	1.78	0.09	0.03	<LOQ	0.02	<LOQ	<LOQ	0.19	0.07	<LOQ	<LOQ	0.27	0.09	0.12
Straupe PKS, "Pienotava", Straupe parish, effluent		0.01	0.36	0.05	1.17	<LOQ	<LOQ	<LOQ	<LOQ	<LOQ	<LOQ	0.25	<LOQ	<LOQ	<LOQ	0.12	<LOQ	<LOQ
SIA "Rūjienas siltums", Rūjiena, influent	57.89389, 25.33933	0.01	1.56	1.12	2.49	0.71	0.17	<LOQ	0.02	0.01	<LOQ	0.82	0.26	<LOQ	0.05	2.21	<LOQ	<LOQ
SIA "Rūjienas siltums", Rūjiena, effluent		4.24	6.78	3.58	3.27	1.03	0.12	<LOQ	<LOQ	<LOQ	<LOQ	12.5	0.19	0.19	0.05	4.51	<LOQ	<LOQ
SIA "Salagrīvas ūdens" WWTP, Salacgrīva, influent	57.75813, 24.35434	0.03	1.62	2.36	3.32	2.52	0.25	<LOQ	0.07	<LOQ	<LOQ	0.59	0.56	2.25	1.47	41.78	1.88	<LOQ
SIA "Salagrīvas ūdens" WWTP, Salacgrīva, effluent		0.59	1.85	0.94	6.23	2.27	0.19	<LOQ	<LOQ	<LOQ	<LOQ	0.98	0.08	1.11	0.36	8.40	<LOQ	<LOQ
Turaida village WWTP, influent	57.18226, 24.85028	0.42	2.03	0.41	1.37	0.35	0.31	<LOQ	0.10	0.01	<LOQ	0.61	0.15	0.06	<LOQ	0.74	<LOQ	<LOQ
Turaida village WWTP, effluent		0.10	1.28	0.13	2.24	0.13	0.09	<LOQ	<LOQ	<LOQ	<LOQ	0.49	0.01	0.02	0.03	0.35	0.01	<LOQ
Līgatne biological WWTP, influent	57.23633, 25.03807	0.01	1.17	0.37	2.78	0.47	0.09	0.05	0.18	<LOQ	<LOQ	0.50	0.02	<LOQ	0.00	0.46	<LOQ	<LOQ
Līgatne biological WWTP, effluent		0.03	1.71	0.44	0.99	0.24	0.02	<LOQ	<LOQ	<LOQ	<LOQ	0.55	0.06	0.02	0.05	0.45	<LOQ	<LOQ
Sēlija WWTP, Sēlpils parish, influent	56.52801, 25.61312	0.02	1.46	0.24	0.91	0.17	0.05	0.02	0.04	<LOQ	<LOQ	0.26	0.01	0.41	<LOQ	0.61	0.00	<LOQ
Sēlija WWTP, Sēlpils parish, effluent		0.02	1.72	0.79	1.20	0.18	0.03	0.02	0.03	<LOQ	<LOQ	0.30	0.01	0.43	<LOQ	0.75	<LOQ	<LOQ
Ozolmuiža village, WWTP BIO II 300, Ozolmuiža parish, influent	56.49696, 27.27792	<LOQ	1.08	0.07	0.62	0.08	0.04	0.02	0.01	<LOQ	<LOQ	0.13	0.00	0.38	0.00	0.76	0.01	<LOQ
Ozolmuiža village, WWTP BIO II 300, Ozolmuiža parish, effluent		0.01	1.76	0.13	0.32	0.09	0.02	0.02	0.02	<LOQ	<LOQ	0.13	<LOQ	0.40	<LOQ	0.51	<LOQ	<LOQ
SIA "Gulbenes nami", Pilskalna village WWTP, Gulbene district, influent	57.28817, 26.78724	0.01	1.38	0.38	0.65	0.11	0.04	<LOQ	0.04	<LOQ	<LOQ	0.22	0.02	0.43	0.01	0.77	<LOQ	0.01
SIA "Gulbenes nami", Pilskalna village WWTP, Gulbene district, effluent		0.01	1.70	0.41	1.02	0.05	0.04	0.01	0.04	<LOQ	<LOQ	0.47	0.03	0.43	0.01	0.84	<LOQ	<LOQ
Skriveri SAC "Ziedugravas" WWTP ASD BX −100, Skriveri district, influent	56.63707, 25.11434	0.01	0.89	0.11	0.56	0.17	0.15	0.00	0.02	<LOQ	<LOQ	0.15	0.01	0.43	<LOQ	1.39	<LOQ	0.02
Skriveri SAC "Ziedugravas" WWTP ASD BX −100, Skriveri district, effluent		<LOQ	1.09	0.13	0.39	0.19	0.09	0.01	0.04	<LOQ	<LOQ	0.34	0.01	0.43	<LOQ	1.04	<LOQ	<LOQ
Žīguri village, WWTP BIO KRB 60, Žīguru parish, influent	57.29366, 27.67204	1.11	2.13	1.86	2.67	1.07	0.15	<LOQ	<LOQ	<LOQ	<LOQ	1.68	<LOQ	0.02	<LOQ	2.09	<LOQ	<LOQ
Žīguri village, WWTP BIO KRB 60, Žīguru parish, effluent		0.68	1.00	1.52	2.11	0.94	0.11	<LOQ	<LOQ	<LOQ	<LOQ	1.77	0.06	0.11	<LOQ	1.35	<LOQ	<LOQ
Vilaka WWTP BIO - 240, Vilaka, influent	57.18795, 27.6825	0.03	0.67	4.04	1.32	9.28	0.07	0.02	0.07	0.19	0.13	0.62	0.08	<LOQ	<LOQ	0.89	<LOQ	0.16
Vilaka WWTP BIO - 240, Vilaka, effluent		0.27	0.86	3.25	1.98	10.2	0.04	<LOQ	<LOQ	<LOQ	<LOQ	0.75	0.11	<LOQ	<LOQ	0.97	0.08	0.02
Ludza, WWTP BIO-2800, Isnaudas parish, influent	56.48444, 27.83591	6.34	7.45	5.89	10.1	4.01	0.60	<LOQ	<LOQ	0.03	0.18	1.75	<LOQ	0.11	<LOQ	2.29	<LOQ	<LOQ
Ludza, WWTP BIO-2800, Isnaudas parish, effluent		4.35	5.57	1.53	4.18	1.57	0.19	<LOQ	<LOQ	<LOQ	<LOQ	1.70	<LOQ	0.07	<LOQ	1.78	<LOQ	<LOQ
Nirzas village, WWTP BIO M 27,5 × 2, Nirzas parish, influent	56.39374, 27.90853	<LOQ	0.84	0.31	0.98	1.08	0.08	<LOQ	<LOQ	<LOQ	<LOQ	0.11	<LOQ	<LOQ	<LOQ	0.18	<LOQ	<LOQ
Nirzas village, WWTP BIO M 27,5 × 2, Nirzas parish, effluent		0.34	0.94	0.59	2.23	1.24	0.25	<LOQ	<LOQ	<LOQ	<LOQ	0.33	<LOQ	<LOQ	0.02	0.65	<LOQ	<LOQ
Raipole village WWTP BIO 50 (Mechanical), "Lupinas", Kusneri, Nirzas parish, influent	56.37307, 27.89586	0.01	0.05	0.25	1.28	0.16	0.20	0.03	<LOQ	<LOQ	<LOQ	0.06	<LOQ	<LOQ	<LOQ	0.34	<LOQ	2.17
Raipole village WWTP BIO 50 (Mechanical), "Lupinas", Kusneri, Nirzas parish, effluent		0.01	<LOQ	0.10	0.07	0.04	<LOQ	<LOQ	<LOQ	<LOQ	<LOQ	0.07	<LOQ	<LOQ	<LOQ	<LOQ	<LOQ	<LOQ
SIA "Madonas ūdens", Liezēres WWTP BIO-DRY-S-45, Liezēre, Madona district, influent	57.03367, 26.05236	0.08	0.20	0.18	0.41	0.08	<LOQ	<LOQ	<LOQ	<LOQ	<LOQ	0.08	<LOQ	0.07	<LOQ	0.80	<LOQ	0.12
SIA "Madonas ūdens", LiezēresWWTP BIO-DRY-S-45, Liezēre, Madona district, effluent		0.52	1.31	0.79	1.42	0.33	0.11	<LOQ	<LOQ	<LOQ	<LOQ	0.51	0.07	<LOQ	<LOQ	1.56	<LOQ	<LOQ
SIA "Mālpils piensaimnieks" WWTP, Mālpils region, influent	57.00842, 24.95752	<LOQ	<LOQ	0.12	0.23	0.02	0.01	<LOQ	<LOQ	<LOQ	<LOQ	<LOQ	<LOQ	<LOQ	<LOQ	0.25	<LOQ	0.03
SIA "Mālpils piensaimnieks" WWTP, Mālpils, Mālpils region, effluent		<LOQ	<LOQ	0.18	0.95	0.06	0.03	<LOQ	<LOQ	<LOQ	<LOQ	<LOQ	<LOQ	<LOQ	<LOQ	<LOQ	<LOQ	0.02
SIA "Saltavots" WWTP, "Jaunlorupes", Sigulda region, influent	57.14387, 24.93476	0.19	2.39	0.68	2.11	0.21	0.15	<LOQ	<LOQ	<LOQ	<LOQ	2.17	0.08	<LOQ	<LOQ	0.85	<LOQ	<LOQ
SIA "Saltavots" WWTP, "Jaunlorupes", Sigulda region, effluent		<LOQ	<LOQ	0.45	3.92	0.14	0.03	<LOQ	<LOQ	<LOQ	<LOQ	0.42	0.00	<LOQ	<LOQ	0.21	0.01	<LOQ
SIA "Ādažu ūdens" "Centre treatment plant", Ādažu district, influent	57.06847, 24.3394	0.08	3.22	1.36	6.45	0.36	0.28	<LOQ	<LOQ	<LOQ	<LOQ	0.30	0.01	<LOQ	<LOQ	<LOQ	<LOQ	0.03
SIA "Ādažu ūdens" "Centre treatment plant", Ādažu district, effluent		2.61	5.33	1.30	6.83	0.29	0.10	<LOQ	<LOQ	<LOQ	<LOQ	4.23	<LOQ	<LOQ	<LOQ	0.26	<LOQ	<LOQ
JSC "Olainfarm", production WWTP, Olaine region, influent	56.7892, 23.95962	<LOQ	<LOQ	2.48	1.64	0.46	0.04	<LOQ	<LOQ	<LOQ	<LOQ	0.49	<LOQ	<LOQ	<LOQ	<LOQ	<LOQ	<LOQ
JSC "Olainfarm", production WWTP, Olaine region, effluent		1.00	1.76	8.41	2.07	0.94	0.05	<LOQ	<LOQ	<LOQ	<LOQ	0.76	<LOQ	<LOQ	<LOQ	0.18	<LOQ	0.03
PSIA "Vangažu avots" WWTP "Kārļzemnieki", Inčukalns district, influent	57.09544, 24.65032	0.01	0.66	0.68	6.36	0.38	0.36	0.01	0.02	<LOQ	0.00	0.40	<LOQ	<LOQ	<LOQ	0.29	<LOQ	0.02
PSIA "Vangažu avots" WWTP "Kārļzemnieki", Inčukalns district, effluent		0.00	0.52	0.43	1.90	0.20	0.20	0.01	<LOQ	<LOQ	<LOQ	0.18	0.00	<LOQ	<LOQ	0.49	<LOQ	<LOQ
JSC "Jaunpils pienotava" WWTP, influent	56.72998, 23.01658	0.30	0.98	0.04	1.05	0.15	0.02	<LOQ	0.11	<LOQ	<LOQ	0.40	0.08	0.04	<LOQ	1.08	0.15	0.48
JSC "Jaunpils pienotava" WWTP, effluent		0.41	0.97	<LOQ	0.37	0.06	0.01	<LOQ	0.02	<LOQ	<LOQ	0.13	0.09	<LOQ	0.02	1.23	0.09	0.08
Valdeku village WWTP influent	56.99956, 22.77259	0.24	0.65	0.17	0.95	0.29	0.40	0.75	1.40	1.79	1.06	0.42	0.18	0.17	<LOQ	1.84	0.58	<LOQ
Valdeku village WWTP effluent		1.23	2.52	0.79	2.49	0.39	0.24	<LOQ	<LOQ	<LOQ	<LOQ	0.91	0.05	0.79	0.08	3.03	<LOQ	<LOQ
Kazdanga WWTP "Avenes", Aizpute region, influent	56.73262, 21.7328	0.05	0.56	0.05	4.07	0.28	0.06	<LOQ	0.03	<LOQ	<LOQ	0.28	0.57	0.05	<LOQ	2.46	0.44	<LOQ
Kazdanga WWTP "Avenes", Aizpute region, effluent		0.14	0.62	0.13	1.35	0.27	0.02	<LOQ	0.01	<LOQ	<LOQ	0.53	0.78	0.13	0.49	1.96	<LOQ	<LOQ
SIA "Durbes KS" WWTP Raibenieki, Durbe district, influent	56.62798, 21.49162	0.12	0.52	<LOQ	1.06	0.29	0.09	<LOQ	0.01	<LOQ	<LOQ	0.39	1.38	<LOQ	<LOQ	6.21	0.33	<LOQ
SIA "Durbes KS" WWTP Raibenieki, Durbe district, effluent		0.26	1.21	0.35	7.57	0.19	0.08	<LOQ	0.02	<LOQ	<LOQ	0.43	0.04	0.35	0.06	1.43	<LOQ	<LOQ
WWTP "Cīruļi", Valdgale parish, influent	57.35444, 22.37565	0.45	2.13	0.29	2.51	0.66	<LOQ	<LOQ	0.04	<LOQ	<LOQ	0.98	1.99	0.29	0.23	4.30	0.25	<LOQ
WWTP "Cīruļi", Valdgale parish, influent		0.39	2.68	0.06	7.07	0.67	<LOQ	0.01	0.01	0.04	0.02	0.72	2.69	0.06	<LOQ	5.46	<LOQ	<LOQ
WWTP, Viduskurzeme AAO, Brocēnu region, influent	56.73475, 22.63573	1169	1528	406	801	59.4	32.8	1.88	1.19	0.99	0.74	532	5.33	406	5.02	201	<LOQ	<LOQ
WWTP, Viduskurzeme AAO, Brocēnu region, effluent		1115	1574	240	237	19.6	4.01	0.21	0.20	<LOQ	<LOQ	346	4.14	240	0.00	64.8	0.00	0.00
SIA "Madonas ūdens", Laudona WWTP BI0-KRD-17, Madona District, influent	56.70736, 26.19354	0.01	3.12	0.83	1.59	0.28	0.10	<LOQ	0.01	<LOQ	0.00	0.47	0.01	0.44	0.01	1.11	0.09	0.09
SIA "Madonas ūdens", Laudona WWTP BI0-KRD-17, Madona District, effluent		1.18	3.68	1.82	7.54	0.71	0.38	0.01	<LOQ	<LOQ	<LOQ	0.26	<LOQ	<LOQ	<LOQ	1.57	<LOQ	0.03
SIA "Olaines ūdens un siltums", municipal WWTP, Olaine district, influent	56.78967, 23.92957	0.76	2.78	2.46	3.95	0.75	0.24	0.10	<LOQ	<LOQ	<LOQ	8.48	0.06	0.06	<LOQ	0.95	<LOQ	0.02
SIA "Olaines ūdens un siltums", municipal WWTP, Olaine district, effluent		0.58	2.49	2.53	3.55	0.44	0.05	<LOQ	<LOQ	<LOQ	<LOQ	13.3	0.03	0.02	<LOQ	0.18	<LOQ	<LOQ
SIA "Gulbenes nami", Lizuma village WWTP, Gulbene district, influent	57.21902, 26.36204	<LOQ	0.87	0.16	0.48	0.10	<LOQ	0.01	0.05	<LOQ	<LOQ	0.43	0.08	0.50	<LOQ	0.64	<LOQ	<LOQ
SIA "Gulbenes nami", Lizuma village WWTP, Gulbene district, effluent		1.07	1.85	0.37	0.56	0.12	0.08	0.01	<LOQ	<LOQ	<LOQ	0.43	<LOQ	<LOQ	0.04	0.38	<LOQ	<LOQ
JSC "Rankas piens", Ranka WWTP, Gulbene district, influent	57.17309, 26.76197	0.31	0.59	0.30	1.06	0.05	0.02	<LOQ	<LOQ	<LOQ	<LOQ	0.36	<LOQ	<LOQ	0.09	0.12	<LOQ	0.07
JSC "Rankas piens", Ranka WWTP, Gulbene district, effluent		0.02	1.38	0.24	0.64	0.04	0.03	<LOQ	0.04	<LOQ	<LOQ	0.32	0.02	0.43	0.00	0.71	<LOQ	<LOQ
Abavnieki village WWTP influent	56.97438, 22.93197	0.33	0.63	0.08	1.59	0.38	<LOQ	0.01	0.04	0.02	<LOQ	1.22	1.76	0.08	0.70	20.5	<LOQ	<LOQ
Abavnieki village WWTP effluent		0.66	1.27	0.38	1.04	0.10	0.04	<LOQ	<LOQ	<LOQ	<LOQ	0.92	0.04	<LOQ	<LOQ	0.95	<LOQ	<LOQ
Popes WWTP "Pope", Popes Parish, influent	57.44606, 21.87923	5.70	8.42	0.25	10.4	1.91	1.37	0.21	0.07	0.49	0.55	2.95	0.17	0.25	0.20	7.34	<LOQ	<LOQ
Popes WWTP "Pope", Popes Parish, effluent		0.49	1.37	0.44	0.79	0.20	0.11	0.07	<LOQ	<LOQ	<LOQ	0.44	0.03	0.03	0.08	0.47	0.03	0.27
SIA "L LIKO FARM", Strazdi, Saldus district, influent	56.59974, 22.41208	0.01	<LOQ	1.03	2.03	0.21	0.08	0.02	<LOQ	<LOQ	<LOQ	0.38	0.04	<LOQ	<LOQ	0.60	<LOQ	<LOQ
SIA "L LIKO FARM", Strazdi, Saldus district, effluent		0.56	1.40	0.94	4.86	0.28	0.07	0.01	0.03	<LOQ	<LOQ	0.79	0.13	0.94	<LOQ	1.85	<LOQ	<LOQ

## 2 Experimental Design, Materials and Methods

Wastewater aliquots were collected into 1 L brown coloured glass bottles and transferred to the laboratory at + 4 °C. Samples were stored at +4 °C and treated within seven days of receipt.

Selected PFAS were analysed according to the previously published method with minor modifications in order to extend the scope of the target analytes [1]. 250 mL sample aliquots were spiked with ^13^C-isotopically labeled PFAS surrogates (5 ng of each component) served as internal standards and then 100 µL of formic acid was added to each sample. Samples were loaded at a flow rate of ∼ 5 mL min^−1^ on the weak anion-exchange SPE columns (Strata-X-AW 200 mg/3 mL (Phenomenex, Torrance, CA, USA)), which were previously pre-washed with 3 mL of 1% NH_4_OH in MTBE/MeOH (90:10, v/v), 3 mL of MeOH, and 3 mL of ultra-pure water. After the sample loading, the SPE cartridges were washed with 1 mL of 2% formic acid and 2 mL of MeOH, and after drying the cartridges for 30 min under vacuum, the analytes were eluted with 7 mL of 1% NH_4_OH in MTBE/MeOH (90/10, v/v) to a 10 mL glass vial. Eluted extracts were evaporated to dryness under a gentle stream of nitrogen at 30 °C, reconstituted with MeOH (100 µL) and subjected to the HPLC-Orbitrap-MS analysis. Prepared extracts were analysed within three days of sample preparation. Instrumental analysis was performed using HPLC-Orbitrap-MS operated in parallel reaction monitoring (PRM) mode, with detection of negative ions. Quantitation was carried out using isotope dilution and internal standardisation method with selected ^13^C-labeled surrogates. The results from real samples were corrected by subtracting the PFAS concentrtions of the procedural blanks (see Supporting Information) analysed in each sample sequence. Method accuracy was controlled by the analysis of certified reference material (CRM) Standard Reference Material 2781 Domestic Sludge (NIST, Gaithersburg, MD, USA), which was analysed in each sample run in the frame of the current study. Boxplots were constructed for datasets as follows: the horizontal line within the box represents the distribution median. The ends of the box represent the 25th and 75th percentiles, while the interquartile range (IQR) is the difference between the 25th and 75th percentiles. The lines that extend from each end are whiskers, from the ends of the box to the outermost data point that falls within the distances computed as follows: 1st quartile−1.5 × (IQR) 3rd quartile +1.5 × (IQR).

## Ethics Statement

Not applicable*.*

## CRediT authorship contribution statement

**Dzintars Zacs:** Methodology, Writing – original draft, Visualization. **Elina Pasecnaja:** Methodology, Software, Data curation. **Vadims Bartkevics:** Supervision, Writing – review & editing.

## Declaration of Competing Interest

The authors declare that they have no known competing financial interests or personal relationships that could have appeared to influence the work reported in this paper.

## Data Availability

Data on occurrence of perfluoroalkyl substances in influents and effluents collected from different wastewater treatment plants in Latvia (Original data) (https://figshare.com/).

## Appendix Supplementary materials

Image, application 1

## References

[1] Zacs D., Bartkevics V. (2016). Trace determination of perfluorooctane sulfonate and perfluorooctanoic acid in environmental samples (surface water, wastewater, biota, sediments, and sewage sludge using liquid chromatography–Orbitrap mass spectrometry. J. Chromatogr. A.

